# Supplementary material for: Pelvic neural injuries and acute voiding changes in rat models of radical hysterectomy
Source: PLoS One. 2024 Oct 11;19(10):e0311559. doi: 10.1371/journal.pone.0311559 (PMC11469490; doi:10.1371/journal.pone.0311559)
Supplement: Attachment — Submitted filename: PONE response 20240714WYP.docx [file pone.0311559.s002.docx]

July 14, 2024

Dear Editor,

Thank you for your letter and advice regarding the review and revision of our manuscript entitled “Pelvic neural injuries and acute voiding changes in rat models of radical hysterectomy”. We have addressed all the comments raised by the reviewers and have revised the manuscript accordingly. A point-to-point reply is included as follows. We appreciate all the reviewers, and thanks for their comments and great work.

We would like to re-submit the revised manuscript for your consideration. We hope that the revision is acceptable, and we look forward to hearing from you soon.

Best Regards!

Yours sincerely,

Yan-peng Wang

Department of Gynecology, Zhejiang Provincial People's Hospital, 158 Shangtang Road, Hangzhou, Zhejiang Province, China, 310014. Tel: +86-150-5717-2973; E-mail: [wangyanpeng@hmc.edu.cn](mailto:wangyanpeng@hmc.edu.cn)

Reviewer #1: It is an interesting work, the authors spend a remarkable effort to obtain their results, the result was well presented, some remarques required a major revision

In abstract

Add a brief explanation to compare the various terms of types A, B1, C1 and C2.

Add (using immunohistochemistry) in methodology.

It is better to use passive tense rather using (We established …..)

In introduction

Line 49: Add a paragraph to compare the various terms of types A, B1, C1 and C2.

Line 54: Add a paragraph to explain the normal anatomy of pelvic plexus in human and to justify the use of rat model with references.

The paragraph between lines 59 and 61 need a suitable reference.

Line 62: (Not only did we show the structural) needs lingual revision, native speaker revision is demanded.

**Response:** Thank you for your valuable advice. We have revised the manuscript according to the reviewer’s suggestion.

In results

It is not clear whether the loose of rats in B1 and C1 was related to the operation complication? Please clarify.

**Response**: Thank you for your valuable advice. We have added this information in the section of “Experimental design”: Besides, one rat in type B1 group and two rats in type C1 group died 2 to 3 days after operation due to operation complication, and additional rats were supplemented.

Line 98: Adding schematic drawing to explain the different types of procedure with location of nerves will help to explain.

Line 171: Adding samples of images of 2.5 and 2.6 procedures could help to explain.

Line 195: Add more information about the primary and secondary antibodies and the action of Pgp9.5. with reference>

**Response:** The reviewer’s comment is well appreciated. We have revised the manuscript, figure 1 and figure 3 according to the advices.

In discussion:

The clinical application according to the results of this work was not obvious, is it the standardizing of the model or the application in surgical technique in human.

**Response:** The reviewer’s comment is well appreciated. We have added some comments on this topic at the end of the first paragraph in the discussion section.

Reviewer #2: The author describes the establishment of a new animal model mimicking neurogenic bladder dysfunction induced by pelvic nerve injury following radical hysterectomy. The new rat model was characterized by the severity of bladder dysfunction being graded by surgical radicality, which is interesting. However, this manuscript still has some points to be resolved, as follows:

Major

1) Several surgical procedures have been developed to create a stratified neurogenic bladder in rats mimicking UAB followed by total hysterectomy. How should the author decide what millimeters of tissue from cervices should be dissected in each rat model?

**Response:** The reviewer’s comment is well appreciated. The average vaginal length of women is approximately 10 cm, and the length of vaginal resection of 0~10mm, at least 10mm, and 15-20mm in human RH of types A, B and C RH respectively^1^. The average vaginal length of adult SD rats is approximately 15 mm; accordingly, the length of vaginal resection we used is minimal, 1-2mm, 2-3mm and 3mm for types A, B, C1 and C2 RH in this study.

2) The author mentioned that residual urine was measured by pressing the lower abdomen (P.9, line 167). Is this reliably reproducible?

**Response:** The reviewer’s comment is well appreciated. A more accurate method of measuring residual urine is bladder intubation after anesthesia, which is rarely used. Abdominal compression is a simple, noninvasive method that is comparable to ultrasound measurements in accuracy^2^, although it may result in a small amount of urine remaining in the bladder. Our data from the same animal at different time points also showed the stability of the measured values.

3) All C2N and C2 rats exhibited NVCs in the CMGs despite being subjected to severe pelvic nerve injury by wider resection. Why? Discuss the issue (P.12, line 240).

**Response:** The reviewer’s comment is well appreciated. In fact, frequent nonvoiding contraction is not a real contraction but the characteristic of instable detrusor after severe urinary retention and neurogenic bladder. We have added this context in the manuscript: “The loss of neural innervation would impair the sensation, autonomic dilation and contraction of bladder, caused urinary retention and instable detrusor with overactive bladder, characterized by overflow incontinence and NVCs.”

Minor

1) how do you evaluate QOL of rat? ( P.12, line 237)

**Response:** The reviewer’s comment is well appreciated. The “Quality of life” referred to the voiding habit and pattern reflected by spotting test. Thus, we changed “quality of life” to “voiding habit” for accuracy.

References

1.	Querleu D, Morrow CP. Classification of radical hysterectomy. *Lancet Oncol*. Mar 2008;9(3):297-303. doi:10.1016/S1470-2045(08)70074-3

2.	Sartori AM, Schwab ME, Kessler TM. Ultrasound: A Valuable Translational Tool to Measure Postvoid Residual in Awake Rats? *Eur Urol Focus*. Sep 15 2020;6(5):916-921. doi:10.1016/j.euf.2019.11.016
