# Supplementary material for: Pelvic neural injuries and acute voiding changes in rat models of radical hysterectomy
Source: PLoS One. 2024 Oct 11;19(10):e0311559. doi: 10.1371/journal.pone.0311559 (PMC11469490; doi:10.1371/journal.pone.0311559)
Supplement: Attachment — Submitted filename: PONE response 20240917WYP.docx [file pone.0311559.s003.docx]

Sep 17, 2024

Dear Editor,

Thank you for your letter and advice regarding the review and revision of our manuscript entitled “Pelvic neural injuries and acute voiding changes in rat models of radical hysterectomy”. We have addressed all the comments raised by the reviewers and have revised the manuscript accordingly. A point-to-point reply is included as follows. We appreciate all the reviewers, and thanks for their comments and great work.

We would like to re-submit the revised manuscript for your consideration. We hope that the revision is acceptable, and we look forward to hearing from you soon.

Best Regards!

Yours sincerely,

Yan-peng Wang

Department of Gynecology, Zhejiang Provincial People's Hospital, 158 Shangtang Road, Hangzhou, Zhejiang Province, China, 310014. Tel: +86-150-5717-2973; E-mail: [wangyanpeng@hmc.edu.cn](mailto:wangyanpeng@hmc.edu.cn)

**Reviewer #2:** The manuscript has now been revised accordingly. However, the following issues remain to be discussed: In the Discussion section, the authors described “ our findings revealed some clues of neurological damage and voiding dysfunction in type A and B1 RHs,,, “ (line 310). In contrast, there were no significant differences in voiding functional parameters between the sham and type A groups (Fig.3 and 4). The authors cannot conclude that even a type A RH procedure causes voiding dysfunction. Please answer.

**Response:** The reviewer’s comment is well appreciated. We have revised the manuscript as “our findings revealed some clues of neurological damage and voiding dysfunction in type B1 RH, as manifested by decreased mean area of urine spots and leakage point pressure, and increased collagen volume fraction in affected rats”.
